# Next-generation sequencing profiling of miRNAs in individuals with 22q11.2 deletion syndrome revealed altered expression of miR-185-5p

**DOI:** 10.1186/s40246-024-00625-5

**Published:** 2024-06-13

**Authors:** Anelisa Gollo Dantas, Beatriz Carvalho Nunes, Natália Nunes, Pedro Galante, Paula Fontes Asprino, Vanessa Kiyomi Ota, Maria Isabel Melaragno

**Affiliations:** 1 Genetics Division, Department of Morphology and Genetics, Universidade Federal de São Paulo, São Paulo, Brazil; 2 Department of Biosciences and Medical Biology, University of Salzburg, Salzburg, Austria; 3 Molecular Oncology Center, Hospital Sírio-Libanês, São Paulo, SP Brazil

**Keywords:** 22q11.2 deletion syndrome, miRNA, Next-generation sequencing, miR-185-5p

## Abstract

**Background:**

The 22q11.2 deletion syndrome (22q11.2DS) is a microdeletion syndrome with highly variable phenotypic manifestations, even though most patients present the typical 3 Mb microdeletion, usually affecting the same ~ 106 genes. One of the genes affected by this deletion is *DGCR8*, which plays a crucial role in miRNA biogenesis. Therefore, the haploinsufficiency of *DGCR8* due to this microdeletion can alter the modulation of the expression of several miRNAs involved in a range of biological processes.

**Results:**

In this study, we used next-generation sequencing to evaluate the miRNAs profiles in the peripheral blood of 12 individuals with typical 22q11DS compared to 12 healthy matched controls. We used the DESeq2 package for differential gene expression analysis and the DIANA-miTED dataset to verify the expression of differentially expressed miRNAs in other tissues. We used miRWalk to predict the target genes of differentially expressed miRNAs. Here, we described two differentially expressed miRNAs in patients compared to controls: hsa-miR-1304-3p, located outside the 22q11.2 region, upregulated in patients, and hsa-miR-185-5p, located in the 22q11.2 region, which showed downregulation. Expression of miR-185-5p is observed in tissues frequently affected in patients with 22q11DS, and previous studies have reported its downregulation in individuals with 22q11DS. hsa-miR-1304-3p has low expression in blood and, thus, needs more validation, though using a sensitive technology allowed us to identify differences in expression between patients and controls.

**Conclusions:**

Thus, lower expression of miR-185-5p can be related to the 22q11.2 deletion and *DGCR8* haploinsufficiency, leading to phenotypic consequences in 22q11.2DS patients, while higher expression of hsa-miR-1304-3p might be related to individual genomic variances due to the heterogeneous background of the Brazilian population.

**Supplementary Information:**

The online version contains supplementary material available at 10.1186/s40246-024-00625-5.

## Background

The 22q11.2 deletion syndrome (22q11.2DS) is the most frequent chromosomal microdeletion syndrome, affecting 1 per 3,000 to 1 per 6,000 live births, based on the diagnosis of infants [1] and 1 per 1,000 fetuses [2]. Large paralogous low copy repeats (LCRs), which are more than 96% identical, flanking the 22q11.2 region, facilitate aberrant recombination during meiosis [3]. The most frequent deletion in this region, covering 3 Mb and accounting for approximately 85% of cases, extends across LCR A-D and leads to a haploinsufficiency of ~ 106 genes. The second most frequent deletion (~ 10–12% cases) covers 1.5 Mb, involving LCR A-B and ~ 30 genes [1, 3–5].

This syndrome affects multiple organs, and approximately 180 clinical manifestations have been described, including characteristic facial features, congenital cardiac malformations, immune deficiency, hypocalcemia, velopharyngeal insufficiency, developmental delay, cognitive impairment, and psychiatric disorders [6]. However, the expression of these phenotypes varies between and within families, and the 22q11.2 hemizygosity alone cannot explain the genetic mechanisms of this heterogeneity.

One of the genes lost in the most frequent deletions in 22q11.2 is the *DGCR8 (DiGeorge Syndrome Critical Region 8)*, a core gene for miRNA biogenesis [7] located in the 1.5 Mb region between LCR A-B [8]. A microprocessor complex composed of *DGCR8* and *DROSHA* is responsible for recognizing and excisioning precursor miRNAs (pre-miRNAs) after their transcription. These are exported to the cytoplasm for further processing into mature miRNAs [9]. According to the latest version of the miRBase database (release 22), the primary public repository and online resource for microRNA sequences and annotation, the human genome contains 2654 mature miRNA sequences [10]. Thus, the haploinsufficiency of *DGCR8* can modulate the expression of numerous miRNAs, which are responsible for guiding the cleavage, degradation, and translational repression of their target transcripts involved in a wide range of biological processes [11]. In addition, alterations in the expression of these miRNAs could lead to uncovering previously silenced mutations that are heterogeneous across individuals [12].

Two previous studies revealed the downregulation of the *DGCR8* gene and alterations in the expression of miRNAs in the blood of 22q11DS patients [13, 14]. In their investigation, Sellie et al. (2014) observed the downregulation of various miRNAs in patients’ blood, suggesting that the extent of the dysregulation could be associated with psychiatric, neurocognitive, and immunological manifestations in 22q11DS. De La Morena et al. (2013) demonstrated a high level of miRNA expression variability in patients compared to controls, suggesting that this variability could be related to immunological and cardiac abnormalities in the patients [14]. Of note, the most significant miRNA in the two studies was the miR-185-5p, located in the 22q11.2 region, showing decreased expression in patients compared to controls [13, 14]. However, Sellie et al. (2014) analyzed only selected miRNAs in North American patients’ blood, while de La Morena et al. (2013) used microarray profiling, which has low sensitivity compared to other techniques [15], to evaluate miRNA expression in subjects with Hispanic, Caucasian or African-American ancestry. Therefore, in this study, we aimed to better analyze miRNA profiles of leukocytes in a Brazilian population of patients with 22q11DS, acknowledging the frequent underrepresentation of this admixed population in studies and databases. To achieve this, we used a sensitive technology, next-generation sequencing, which allowed us to observe differences even in lowly expressed miRNAs in blood.

## Methods

### Study design and subjects

We recruited the patients from the Instituto da Criança e do Adolescente do Hospital das Clínicas da Faculdade de Medicina da Universidade de São Paulo (Children’s Institute of the University of São Paulo Medical School - ICr HCFMUSP) and from the Instituto de Assistência Médica ao Servidor Público Estadual (Institute of Medical Assistance for Public State Servants - IAMSPE). We selected 12 22q11.2DS patients carrying 3 Mb deletions, previously identified by MLPA (Multiplex Ligation-dependent Probe Amplification) using SALSA MLPA Probemix P250 DiGeorge (MRC Holland). We previously used chromosomal microarray analysis (CMA) to narrow the breakpoints of their deletions. We compared the miRNA expression profiles of these 12 patients with those of 12 healthy control subjects (without the deletion) matched for age and sex. Inclusion criteria comprised patients between 14 and 35 years old with confirmed ~ 3 Mb deletions through the SNP array.

### Blood collection, RNA isolation, miRNA library preparation, and sequencing

Blood samples were collected from all participants using PAXgene Blood RNA Tubes (BD), and total RNA was isolated using the PAXgene Blood miRNA Kits (Qiagen). All RNA samples had an RNA Integrity Number value above 7 [16]. We prepared the libraries using 100 ng of RNA and the QIAseq miRNA Library Kit (Qiagen), which adds Unique Molecular Identifiers (UMIs) to the sequences, following the manufacturer’s instructions. Library concentrations and quality were measured using Qubit Fluorimeter (Thermo Fisher Scientific), Qubit dsDNA HS Assay Kit, and Agilent 2100 Bioanalyzer (Agilent) with the Agilent High Sensitivity DNA Kit. The libraries were pooled and sequenced (1 × 75) on the NextSeq 500 instrument (Illumina) using the HighOutput kit (75 cycles). The sequencing generated 662,701,888 reads for the 24 samples (27,612,579 ± 11,254,530 reads per sample).

### Data analyses

We downloaded the Fastq files from Illumina Basespace and determined the miRNA counts using miRge3.0 [17], which is implemented in Python, adding the parameters for Qiagen UMI (--qiagenumi -umi 0,12). We chose this pipeline because of its ability to process UMIs to account for PCR duplicates. Briefly, miRge3.0 performs quality control and adapter trimming using Cutadapt (v3.0). Then, it collapses identical reads into a single read and captures the counts in a Pandas data frame. Reads were aligned to miRBase (v22) using Bowtie (v1.3.0), and the output was then imported into R (v4.1.2) for statistical analysis. We performed differential gene expression analysis using the DESeq2 package [18]. We also tested the pipeline described in Potla et al. [19] and found similar results (Supplementary Figure S1), proceeding with the miRge3.0 pipeline. Before running the DESeq function, we filtered out genes with low expression levels, keeping those with five reads or more in three or more samples. We applied the Default parameters and included the group (case or control) as the outcome variable. A p-value < 0.05 adjusted according to the Benjamini-Hochberg method was considered significant.

The expression of differentially expressed miRNAs was verified in other tissues using DIANA-miTED [20], a systematic collection of miRNA expression values derived from the Sequence Read Archive (SRA) and The Cancer Genome Atlas (TCGA). We selected tissues related to the 22q11DS phenotype (blood, aorta, brain, fetal brain, heart, oropharynx, palate, parathyroid gland, and thymus), both datasets (SRA and TCGA), and all diseases, considering all statuses. Supplementary Table S1 contains the expression data accessed from DIANA-miTED, following the specified parameters.

We used miRWalk (http://mirwalk.umm.uni-heidelberg.de/) [21] to predict the target genes of differentially expressed miRNAs. As input, we included the miRBase accession numbers of the mature miRNAs. We filtered only genes with a binding probability > 0.95, based on the TarPmiR algorithm, those with validated interactions according to miRTarBase and with miRNA binding sites in the 5’UTR, coding sequence, or 3’UTR of the gene. All target genes of any of the differentially expressed miRNAs were included in the gene set enrichment analysis using Enrichr (https://maayanlab.cloud/Enrichr/) [22–24] and were focused on the KEGG pathways (http://www.genome.jp/kegg/, release 2021), Reactome pathways (http://www.reactome.org, release 2016) and Gene Ontology gene sets (http://geneontology.org/, release 2021).

## Results

No difference in sex (eight males and four females in each group, 𝟀2 = < 0.001; *p* = 1.000) or age (Mann-Whitney U = 38; *p* = 0.050) was found between groups. Controls were between 16 and 31 years old (median = 18.5; interquartile range (IQR) = 2.0), and cases ages ranged between 14 and 35 years (median = 16.5; IQR = 3.0).

An average of 94.18% (SD = 1.34%) of the total number of good-quality reads was classified as miRNA, whereas 6.13% (SD = 2.95%) were unclassified (Supplementary Figure S2). The remaining reads were other ncRNA (mean = 3.51%, SD = 1.06%), rRNA (mean = 0.97%, SD = 0.40%), tRNA (mean = 0.58%, SD = 0.24%), snoRNA (mean = 0.57%, SD = 0.31%), mRNA (mean = 0.11%, SD = 0.02%) and hairpin miRNAs (mean = 0.07%, SD = 0.06%). We provide a summary of the raw data in Supplementary Table S2.

**Fig. 1:**
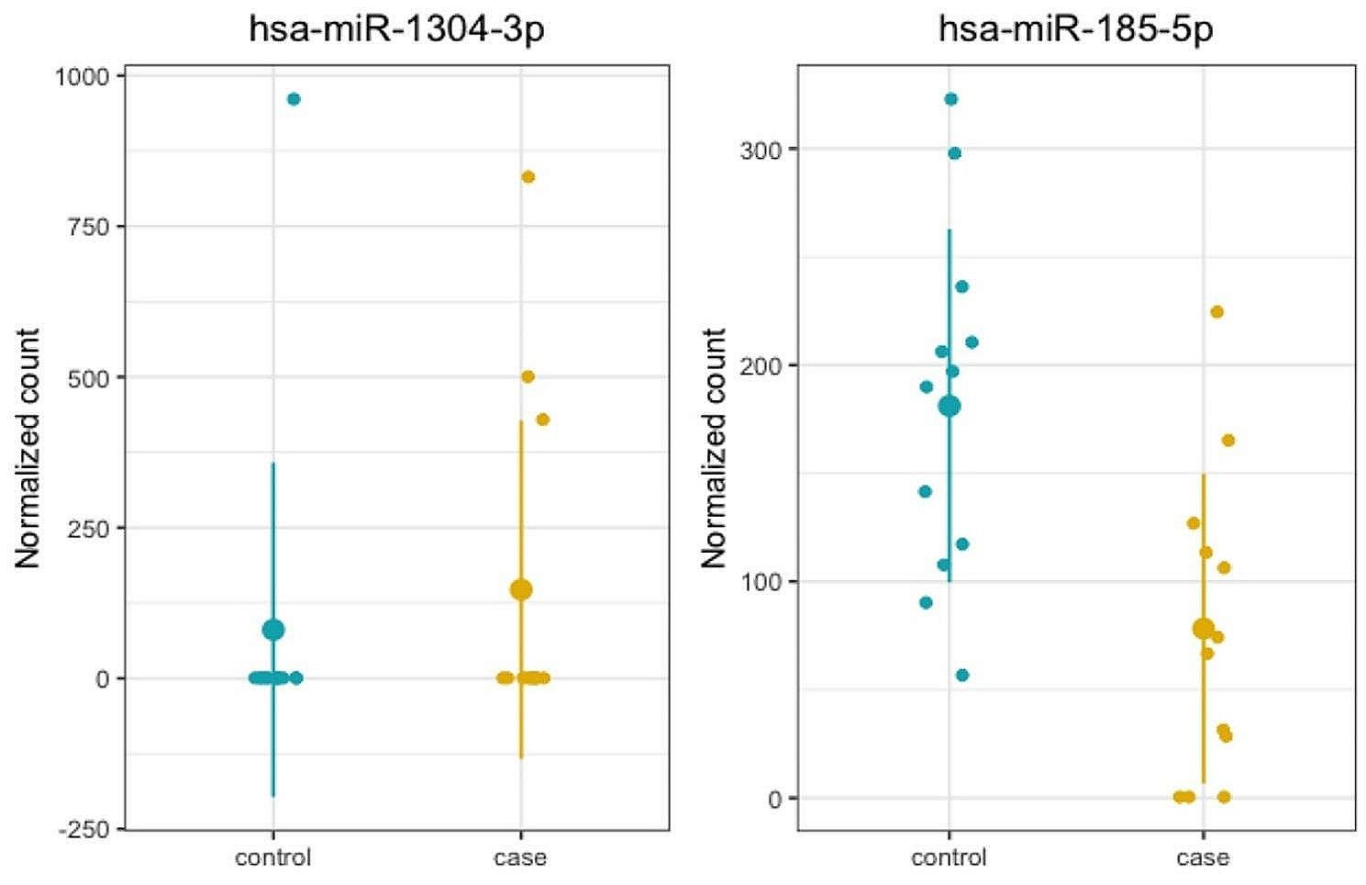
Normalized counts of the differentially expressed miRNAs for each group. hsa-miR-1304-3p was upregulated in cases (log2FC = 24.62; padj = 6.27e-14) and hsa-miR-185-5p, located in the 22q11.2 region, was downregulated (log2FC = -1.51; padj = 7.57e-04)

After removing miRNAs with low read counts, 966 miRNAs remained for the analyses. Principal component analysis (PCA) did not reveal any significant influence of group or sex in the dataset (Supplementary Figure S3). Two miRNAs exhibited differential expression in cases compared to controls: hsa-miR-1304-3p, located in 11q21, was upregulated in cases (log2FC = 24.62; padj = 6.27e-14), and hsa-miR-185-5p, located in the 22q11.2 region, was downregulated (log2FC = -1.51; padj = 7.57e-04) (Fig. 1; Table 1, Supplementary Figures S4 and S5). Although hsa-miR-1304-3p does not seem upregulated in cases due to its low expression, only four samples presented any read count (1 control and 3 cases). Thus, the mean normalized read count for cases is higher than controls, and this difference is significant (log2FC = 24.62; padj = 6.27e-14). We describe these results in Supplementary Table S3. Six other miRNAs are transcribed from the region of the typical 3 Mb deletion at 22q11.2: hsa-miR-1306-5p was downregulated in cases, although not significant after adjusting for multiple comparisons (log2FC = -1.59; padj = 0.142; Supplementary Figure S4); hsa-miR-6816-3p showed no significant difference in expression between the groups (log2FC = -0.69; padj = 0.981); hsa-miR-4761 and hsa-miR-1286 have a minimal expression in blood and had low sequencing coverage; and hsa-miR-3618 and hsa-miR-649 are not expressed on peripheral blood. Besides hsa-miR-1304-3p, hsa-miR-185-5p, and hsa-miR-1306-5p, no other miRNA from the 20 most significantly associated with 22q11.2DS (Table 1) could be related to the syndrome.

**Table 1:** DeSeq2 results of the 20 miRNAs most significantly associated with 22q11.2DS

miRNA	baseMean^a^	log2FoldChange^b^	lfcSE^c^	stat^d^	pvalue^e^	padj^f^
hsa-miR-1304-3p	38,70	24,62	2,95	8,36	6,50E-17	**6,27E-14**
hsa-miR-185-5p*	54195,74	-1,51	0,31	4,80	1,57E-06	**0,000757**
hsa-miR-1299	359,61	-3,74	1,02	-3,69	0,000227	0,073181
hsa-miR-4433b-5p	130,04	-1,75	0,50	-3,52	0,000433	0,083623
hsa-miR-3074-5p	86,99	-0,97	0,27	-3,56	0,000374	0,083623
hsa-miR-548p	4,07	-2,22	0,67	-3,32	0,000889	0,141778
hsa-miR-1306-5p*	1690,98	-1,59	0,48	3,28	0,001027	0,141778
hsa-miR-5000-3p	4,43	-1,69	0,56	3,04	0,002365	0,2856
hsa-miR-379-5p	23,62	-1,25	0,42	-2,98	0,002861	0,30708
hsa-miR-629-5p	1900,53	-0,73	0,25	-2,93	0,003337	0,322364
hsa-miR-3187-3p	53,65	-1,52	0,53	-2,89	0,003886	0,341228
hsa-miR-625-5p	289,58	-0,81	0,28	-2,84	0,004541	0,365552
hsa-miR-6783-3p	91,59	1,12	0,40	2,80	0,005082	0,377602
hsa-miR-3120-3p	6,61	-1,31	0,47	-2,77	0,005521	0,380946
hsa-miR-99b-3p	9,46	-2,71	0,99	-2,73	0,006298	0,38309
hsa-miR-4504-3p	31,37	-1,35	0,49	-2,73	0,006345	0,38309
hsa-miR-6806-3p	58,65	0,84	0,32	2,65	0,008081	0,454799
hsa-miR-1307-3p	944,85	-0,97	0,37	-2,63	0,008475	0,454799
hsa-let-7a-3p	179,44	0,79	0,31	2,53	0,011496	0,52883
hsa-miR-579-5p	17,93	-0,75	0,29	-2,53	0,01142	0,52883

Complete results are shown in Supplementary Table 1^**a**^Average expression level across all samples normalized by sequencing depth ^**b**^Effect size estimate ^c^Standard error estimate for the log2 fold change estimate ^d^Wald statistic ^e^Calculated probability ^f^BH adjusted p-values *miRNAs transcribed from the 22q11.2 region.

We identified no correlation between the expression of hsa-miR-185-5p and hsa-miR-1304-3p (*R* = -0.2; *p* = 0.35; Supplementary Figure S6). Moreover, the low expression of miR-1304-3p in the blood is consistent with its expression levels in other tissues. This miRNA also has low expression in the brain, fetal brain, heart, and parathyroid gland tissues, while it seems to have high expression in the oropharynx, palate, thymus, and aorta. miR-185-5p appears to have high expression in all tissues (Supplementary Figure S7).

We identified a total of 325 target genes to be regulated by miR-1304-3p and 185 by miR-185-5p, with five of them being in common between these miRNAs (*FGFR1, GFPT1, MIEF1, POTED*, and *SHISA9*). We report all target genes identified in Supplementary Table S4. Of note, one gene predicted to be regulated by miR-1304-3p, the *ZDHHC8* (located in the 22q11.2 region), was downregulated in 22q11DS cases in a previous study [25].

By performing enrichment analyses with these target genes, for miR-185-5p, we found four KEGG pathways, one Reactome pathway, eight GO-Biological Processes, four GO-Molecular functions, and two Cellular components enriched for these genes (adj *p* < 0.05; Supplementary Table S5). For miR-1304-3p, we found one KEGG pathway, four GO-Biological Processes, and three GO-Molecular functions enriched for its target genes (adj *p* < 0.05; Supplementary Table S5).

## Discussion

In this study, we used next-generation sequencing to verify miRNA changes in 22q11DS patients compared to controls. The Latin-American population presents a highly heterogeneous genetic background due to the history of admixture in the continent. Genetic findings predominantly based on European populations may not necessarily apply to admixed populations. Therefore, studying miRNAs in a Brazilian population is crucial for gaining a deeper genetic understanding of this group and assessing the generalizability of findings from studies conducted in other populations.

Our findings revealed a downregulation of miR-185-5p, located in the deleted region, and an upregulation of miR-1304-3p in the whole blood of patients. Another miRNA transcribed from the 22q11.2 region, miR-1306-5p, was also downregulated; however, we found no significant association after correction for multiple comparisons. Moreover, miR-185-5p appears to present predominantly expression in tissues associated with the 22q11.2DS, such as the heart and brain, with a widely varying expression in blood. In contrast, miR-1304-3p shows low expression levels in the blood but with higher expression in 22q11DS cases. However, it may exhibit higher expression in other tissues related to the 22q11.2DS, such as the oropharynx, palate, thymus, and aorta. Although the DIANA-miTED database contains both healthy and cancer tissues, these findings are consistent with the biology of the syndrome. Furthermore, it is crucial to note the limited availability of databases assessing miRNA expression in these tissues.

Both previous studies that investigated miRNA expression in 22q11DS patients’ blood found a downregulation of miR-185-5p in patients compared to controls [13, 14]. Moreover, five studies [26–30] in 22q11.2 deletion experimental models with available published data of miRNA profiling in brain or model-brain tissue reported only one downregulated miRNA in common: miR-185-5p [31]. The levels of this miRNA in leukocytes also have been correlated with brain volume in 22q11DS cases [13]. In *Dgcr8*+/- mice, restoring miR-185 levels in presynaptic neurons rescued the hippocampal long-term potentiation phenotype, a form of synaptic plasticity underlying learning and memory [27]. Moreover, this miRNA seems to play a role in cardiac processes, particularly in the heart, by modulating calcium-signaling pathways. Predictive analyses indicate close associations between miR-185 targets and pathways related to TGF-β, BAD, and VEGF. These findings suggest that this miRNA may also affect multiple signal transductions within the heart, potentially contributing to cardiac pathogenesis [32]. Congenital heart diseases are highly prevalent in 22q11.2DS patients, ranging between 22 and 84% depending on patients’ ages [33–35], suggesting that the downregulation of miR-185 could be involved in the cardiac phenotypes of 22q11.2DS. miR-185 seems to be associated with schizophrenia [36, 37], and mouse models suggested that miR-185 is one of the most downregulated miRNAs in schizophrenia-related brain regions [26, 37, 38]. Schizophrenia is also a highly prevalent phenotype on 22q11.2DS, with estimates suggesting that one-third of 22q11.2DS patients will have it in their adulthood [39]. miR-185 also seems to be associated with infectious diseases, being one of the top ten most important downregulated miRNAs as possible biomarkers of severity of COVID-19 [40], and shows anti-inflammatory functions through inhibiting *CDC42*, which has a pro-inflammatory role [41]. Therefore, the downregulation of miR-185 could impact the inhibition of *CDC42*, potentially leading to the inflammatory phenotypes observed in 22q11.2DS patients. Immune dysfunction or allergies are present in more than 70% of 22q11.2DS cases [35]. All these findings indicate a pivotal role of miR-185-5p in the syndrome.

Regarding miR-1304-3p, much less is known, probably due to its low expression in many tissues (Supplementary Figure S7), including whole blood [42]. It seems to regulate immune response genes related to intracranial aneurysms [43] and to be implicated with the development of microcephaly, suggesting a significant role for miR-1304-3p in cerebral cortex development [44]. Therefore, differential expression of this miRNA may be related to neurodevelopmental alterations [44]. Interestingly, the most frequently found miR-1304 allele in humans carries a polymorphism in its seed region predicted to regulate genes involved in biological processes and disorders related to central nervous system development and function. These findings suggest that the evolutionary change of miR-1304, compared to the most frequently found allele in Neanderthal and nonhuman primates, may affect human brain functioning [45]. Finally, a predicted binding site for miR-1304 contains a polymorphism (rs3125) in the *HTR2A* serotonin receptor gene, previously associated with endophenotypes for depression [46]. Anxiety and mood disorders are common in 22q11.2DS patients, especially in children and adolescents [47].

Validated miRNA-target interactions predict that miR-1304-3p regulates 325 genes, while miR-185-5p regulates 185 genes. Of note, the *ZDHHC8* (Zinc finger DHHC-type containing 8) gene, located in the 22q11.2 region and identified as downregulated in our previous study [25], is a target of miR-1304-3p. The *ZDHHC8* gene encodes a putative palmitoyltransferase highly expressed in the brain [48]. This gene was associated with numerous brain functions and disorders, such as schizophrenia [49, 50] and brain morphology [51, 52]. Finally, miR1304-3p gene targets also seem to be enriched for herpes simplex virus 1 infection, reflecting the immune deficiency of this syndrome. Among the enriched miR-185-5p target pathways, we can highlight the cellular response to hypoxia (GO:0071456) and cellular responses to stress (Reactome, R-HSA-2,262,752), which could be associated with some of the 22q11DS phenotypes.

Individual genetic variations in target genes for differentially expressed miRNAs in 22q11.2DS could be related to the heterogeneous clinical manifestations present in the syndrome. A recent study has correlated the presence of additional rare copy number variations (CNVs) overlapping one or more miRNA target genes to the presence of schizophrenia in 22q11DS patients [31]. This correlation warrants further exploration across other phenotypes associated with the syndrome. Therefore, the altered expression of miR-185-5p and miR-1304-3p identified in our study might uncover individual silenced mutations and be associated with the variable phenotypic manifestations present in 22q11.2DS patients.

MicroRNAs play a pivotal role in integrating intracellular signals and regulating signaling pathways [53, 54]. Therefore, the dysregulation of specific miRNAs could be involved in the heterogeneous phenotypic expression observed in 22q11.2DS patients [55]. Further investigations are essential to unravel the consequences of these dysregulations and understand how the interactions between affected pathways contribute to the diverse phenotypic expression of 22q11.2DS. Additionally, when combined with other biomarkers, the dysregulation of miRNAs may contribute to a more comprehensive prognosis and earlier diagnosis for 22q11DS patients. Moreover, understanding the implications of these differentially expressed miRNAs holds potential significance for developing novel therapeutic strategies in the future once further studies regarding miRNAs are made.

Saunders et al. (2007) described that many miRNA target sequences are polymorphic and variable among distinct human populations [56]. These polymorphisms may affect gene expression and help to explain phenotypic variability among humans [56]. Due to the admixed nature of the Brazilian population and the small sample size of our study, conclusions derived from this cohort are limited. The matched controls may not entirely reflect the individual genetic singularities of the patients. Since polymorphisms in miRNA targets may affect expression [56], the admixed background of our patients combined with the sample size might explain some individuals’ high expression of miR-1304-3p. Indeed, a recent study identified a single nucleotide polymorphism (SNP) within the miR-1304 stem-loop region (rs2155248), whose minor allele frequency varies among populations [57], and that regulates the maturation and expression of has-miR-1304-3p.

## Conclusions

Although we used a robust technique to detect miRNAs, our study should be interpreted in light of some limitations, particularly the small sample size, lack of replication in other relevant tissues, and validation of the target genes and functional assays. It is worth noting that this is a rare syndrome with multiple clinical manifestations and a known impact on the miRNA profiles. Indeed, applying next-generation sequencing allowed us to detect alterations in genes with low expression in blood, which was not possible in previous studies [13, 14].

In conclusion, we found a downregulation of miR-185-5p and an upregulation of miR-1304-3p in the whole blood of 22q11DS patients. The upregulation of miR-1304-3p might be related to polymorphisms in miRNA targets, while the downregulation of miR-185-5p can be related to the 22q11.2 deletion and may be associated with heterogeneous clinical manifestations in 22q11.2DS patients.

### Electronic supplementary material

Below is the link to the electronic supplementary material.

Supplementary Material 1

Supplementary Material 2

## Data Availability

The genome data generated during this project will be made available upon request. Due to the sensitive nature of genomic information and in accordance with ethical guidelines, access to the data will be granted solely by contacting the corresponding author. Requests for the genome data should include a brief description of the purpose and intended use of the data, along with the necessary assurances of data privacy and confidentiality. The corresponding author will assess the requests on a case-by-case basis and, if approved, provide the necessary data access and guidance to ensure its appropriate utilization.

## Abbreviations

22q11.2DS: 22q11.2 deletion syndrome
DGCR8: DiGeorge Syndrome Critical Region 8
LCRs: Low Copy Repeats
DROSHA: Double-Stranded RNA-Specific Endoribonuclease
Pre-miRNA: Precursor miRNA
MLPA: Multiplex Ligation-dependent Probe Amplification
CMA: Chromosomal Microarray Analysis
UMIs: Unique Molecular Identifiers
SRA: Sequence Read Archive
TCGA: The Cancer Genome Atlas
KEGG: Kyoto Encyclopedia of Genes and Genomes
IQR: Interquartile range
SD: Standard Deviation
PCA: Principal Component Analysis
FGFR1: Fibroblast Growth Factor Receptor 1
GFPT1: Glutamine Fructose-6-Phosphate Transaminase 1
MIEF1: Mitochondrial Elongation Factor 1
POTED: POTE Ankyrin Domain Family Member D
SHISA9: Shisa Family Member 9
ZDHHC8: Zinc finger DHHC-type containing 8
GO: Gene Ontology
TGF-β: Transforming Growth Factor Beta
BAD: Bcl-2-Associated Death Promoter
VEGF: Vascular Endothelial Growth Factor
COVID-19: Coronavirus disease 19
CDC42: Cell Division Cycle 42
HTR2A: 5-hydroxytryptamine receptor 2 A
CNV: Copy Number Variation
