# Supplementary material for: Next-generation sequencing profiling of miRNAs in individuals with 22q11.2 deletion syndrome revealed altered expression of miR-185-5p
Source: Hum Genomics. 2024 Jun 13;18:64. doi: 10.1186/s40246-024-00625-5 (PMC11170780; doi:10.1186/s40246-024-00625-5)
Supplement: Supplementary file 2 — Supplementary Material 2 [file 40246_2024_625_MOESM2_ESM.docx]

**Supplementary Figures**


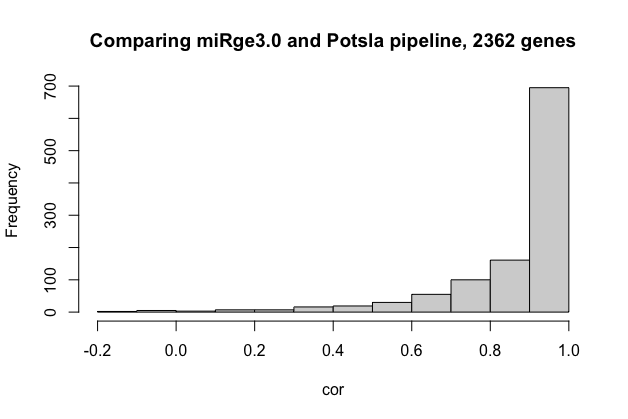


**Supplementary Figure S1.** Frequency of miRNAs exhibiting correlation values between miRge3.0 and Potla pipelines. For example, 695 (out of 2362) miRNAs measured using miRge3.0 had a correlation r > 0.9 compared to the same measured using the Potla pipeline.


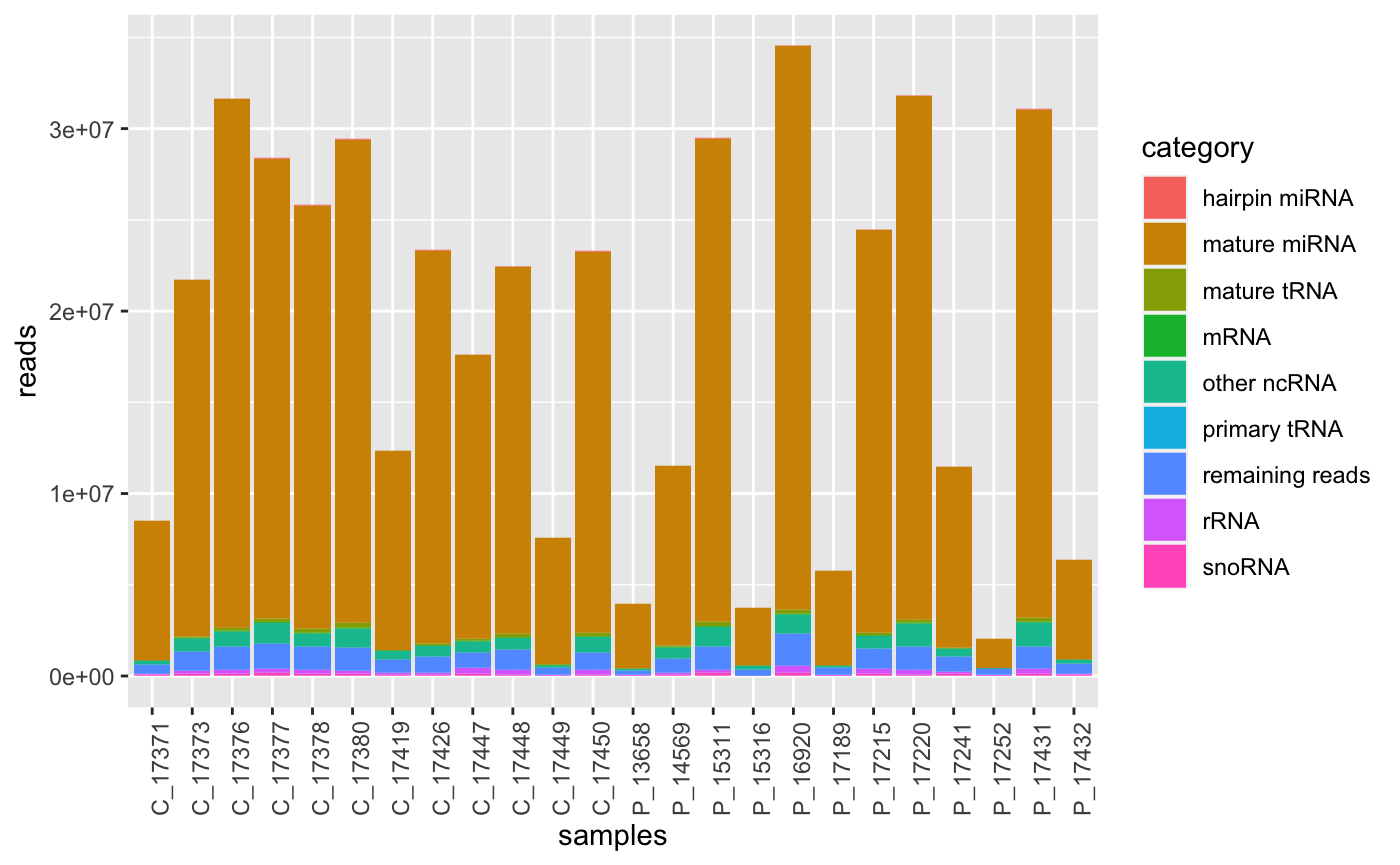


**Supplementary Figure S2:** Number of reads aligned to each dataset category. The majority of reads were from mature miRNAs.

**
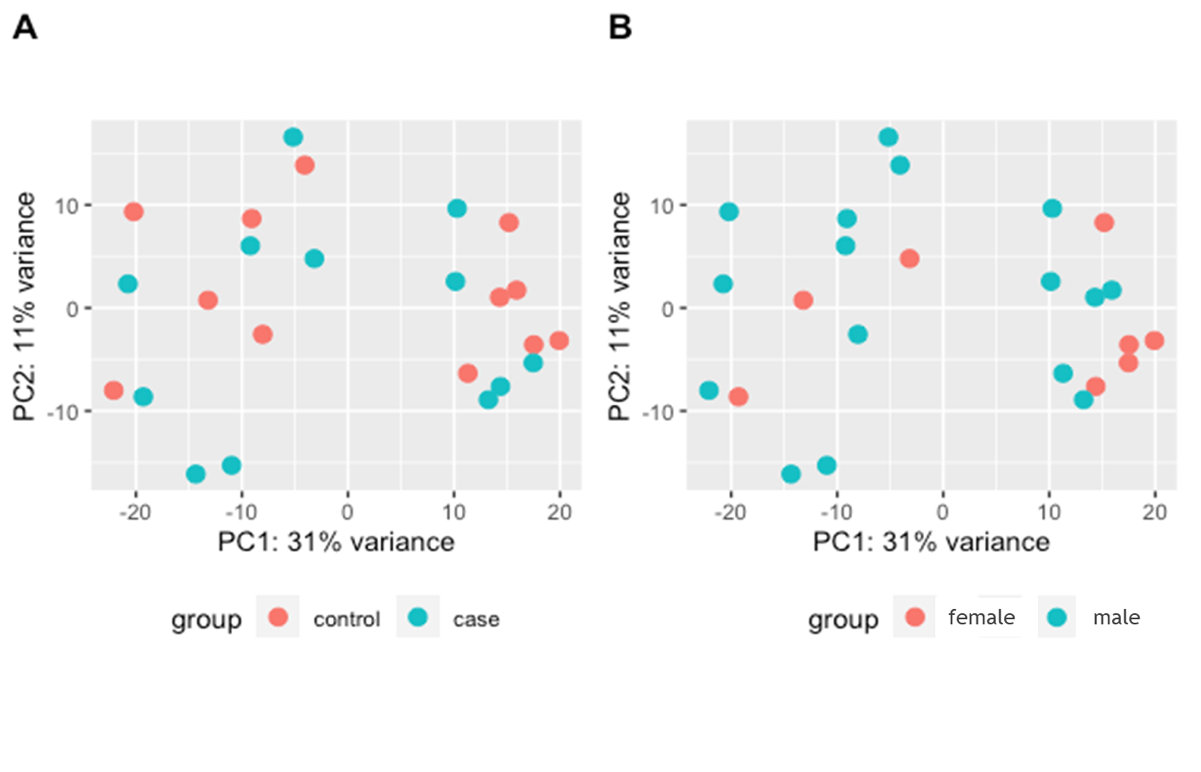
**

**Supplementary Figure S3:** Principal component analysis results, plotting the Principal Component (PC) 1 against PC2 and coloring by group (A) or sex (B).


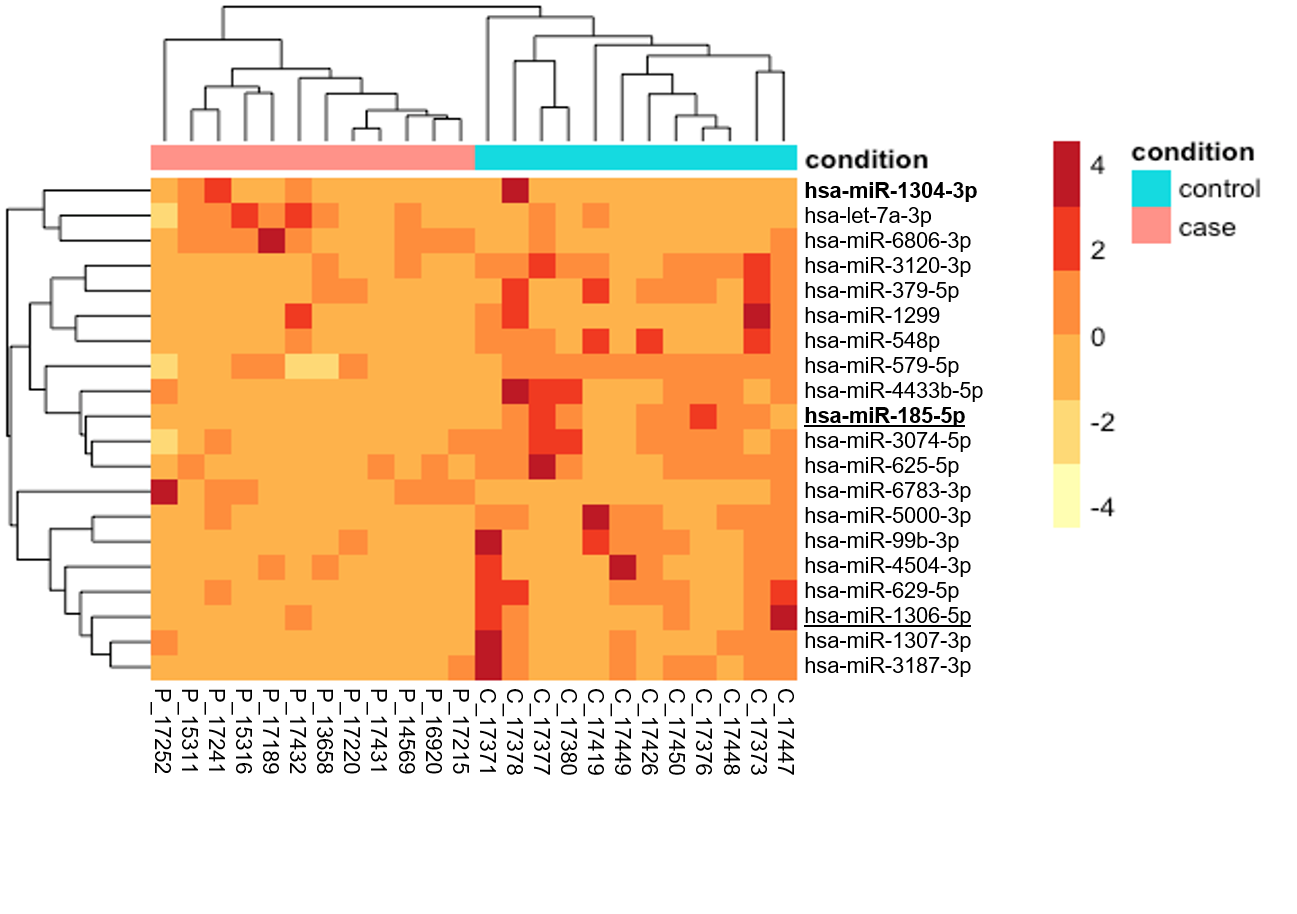


**Supplementary Figure S4:** Heatmap displaying the 20 miRNAs most significantly associated with 22q11.2DS. DESeq2 normalized values were used as input and scaled by row using the pheatmap function. hsa-miR-1304-3p and hsa-miR-185-5p (in bold) have significantly high and low expression, respectively, in patients compared to controls. Of note, hsa-miR-185-5p and hsa-miR-1306-5p (underlined) are transcribed from the 22q11.2 region.


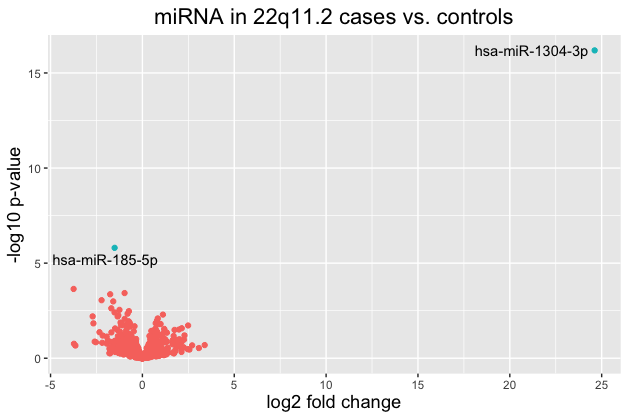


**Supplementary Figure S5:** Volcano plot showing the p-values and fold change comparing 22q11.2DS cases and controls across all the 966 miRNAs assessed. Blue dots represent the significant miRNAs, and pink dots represent the non-significant.


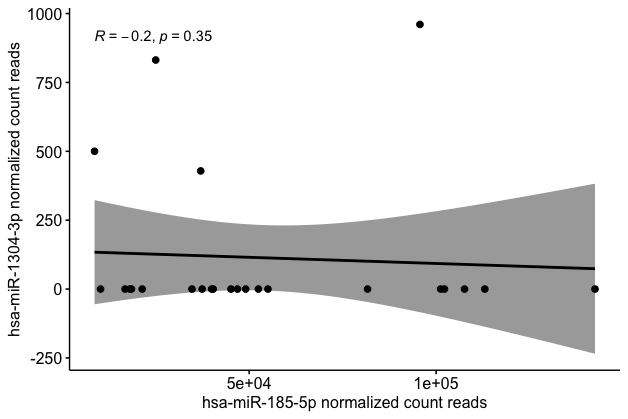


**Supplementary Figure S6:** Scatter plot comparing the normalized counts of hsa-miR-185-5p to hsa-miR-1304-3p. Correlation method: Spearman's rank correlation coefficient.


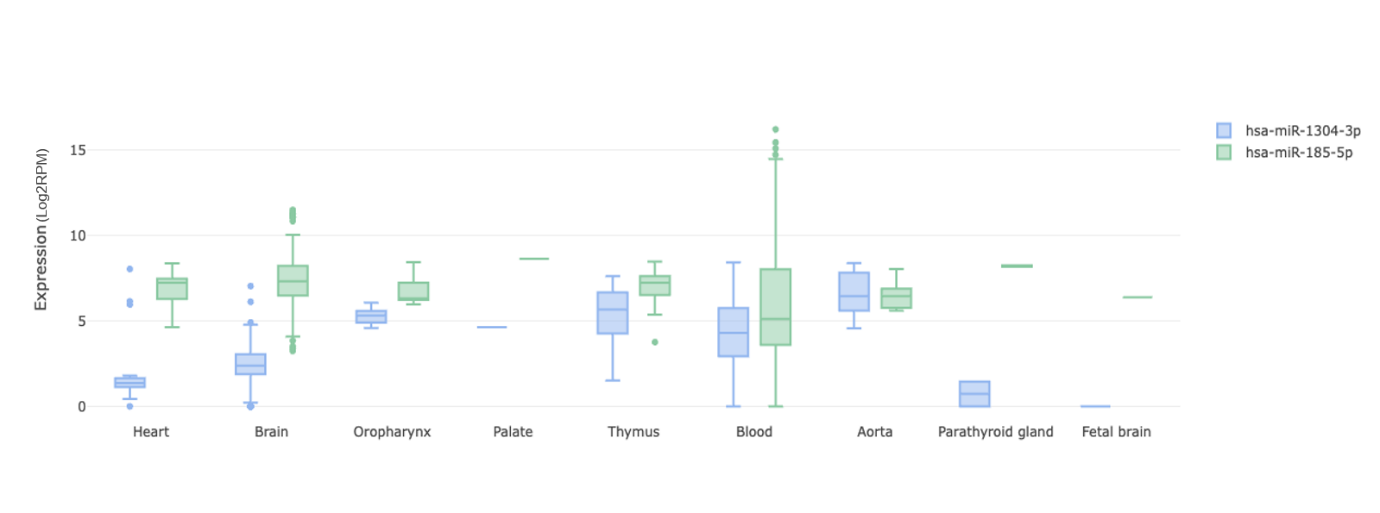


**Supplementary Figure S7:** Boxplots generated by DIANA-miTED display the aggregated expression values (Log2RPM) of the queried miRNAs.
